# Supplementary figures and images for: Development of new mutant alleles and markers for KTI1 and KTI3 via CRISPR/Cas9-mediated mutagenesis to reduce trypsin inhibitor content and activity in soybean seeds
Source: Front Plant Sci. 2023 May 8;14:1111680. doi: 10.3389/fpls.2023.1111680 (PMC10200896; doi:10.3389/fpls.2023.1111680)

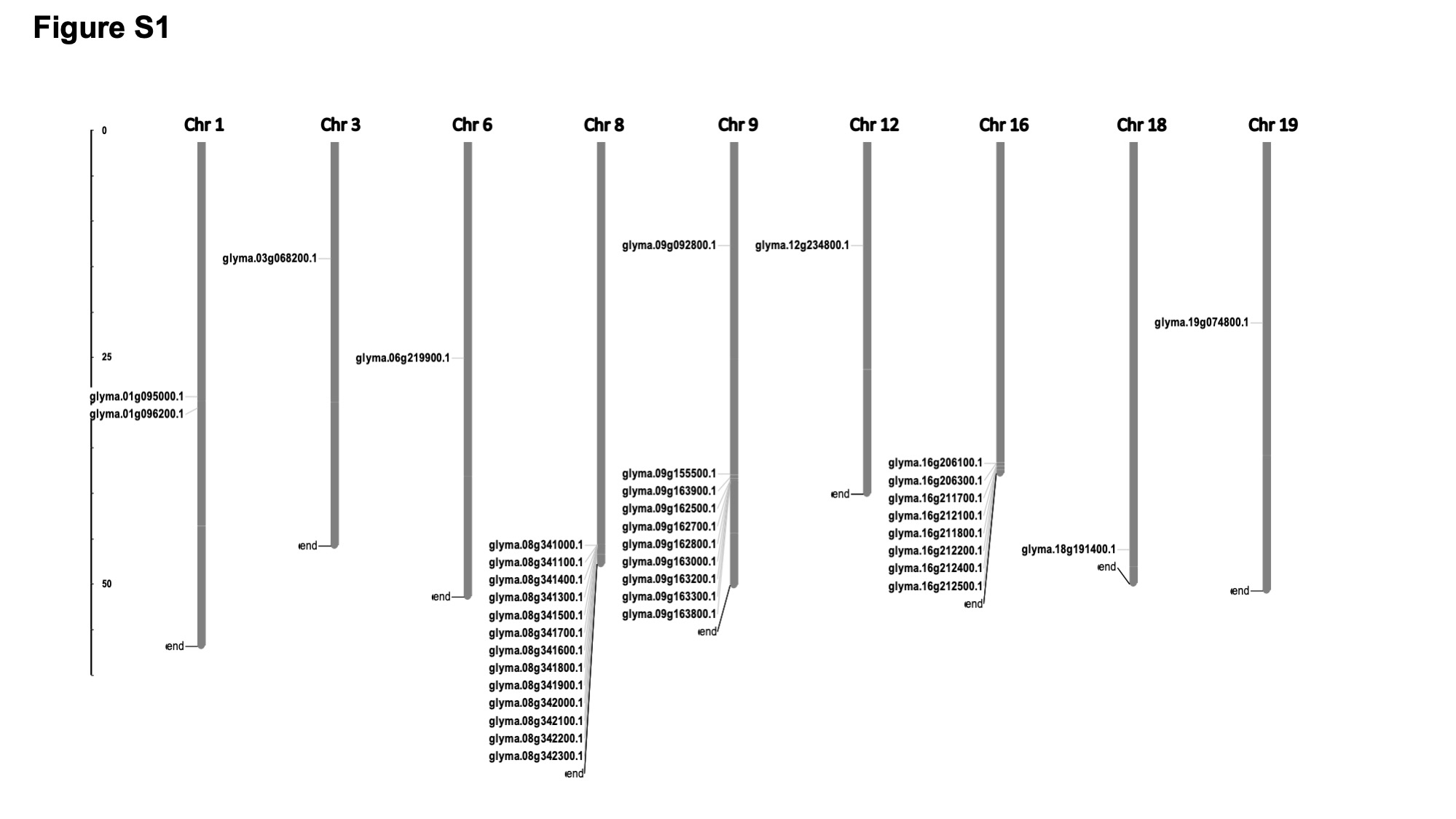

Supplement: Supplementary Figure 1 — Physical mapping of 38 GmKTI genes on soybean 20 chromosomes. The gene map showing locations of KTI genes on soybean chromosomes was made using MapInspect. As displayed in the map, 38 KTI genes are located on 9 out of 20 chromosomes. [file Image_1.jpeg]

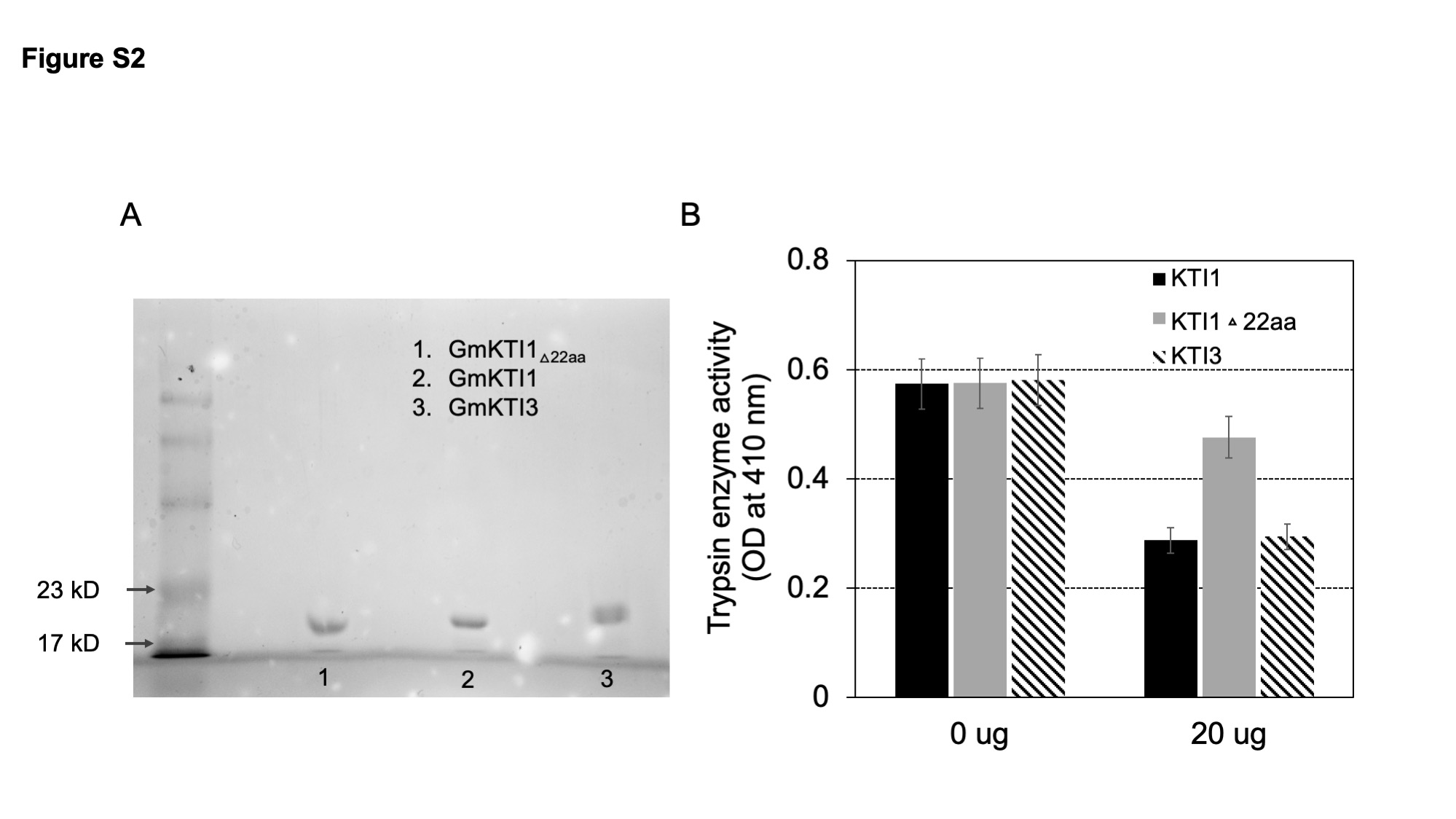

Supplement: Supplementary Figure 2 — SDS-PAGE of purified recombinant proteins and in-frame mutated protein of KTI1Δ22aa nearly lost the TIA. (A) SDS-PAGE was used to assess the purity of three recombinant proteins, KTI1, KTI3, and KTI1Δ22aa. (B) Purified proteins of KTI1 and KTI3, but not KTI1Δ22aa were able to inhibit the trypsin activity in vivo. Experiments were conducted with three technical replicates and obtained similar results. [file Image_2.jpeg]

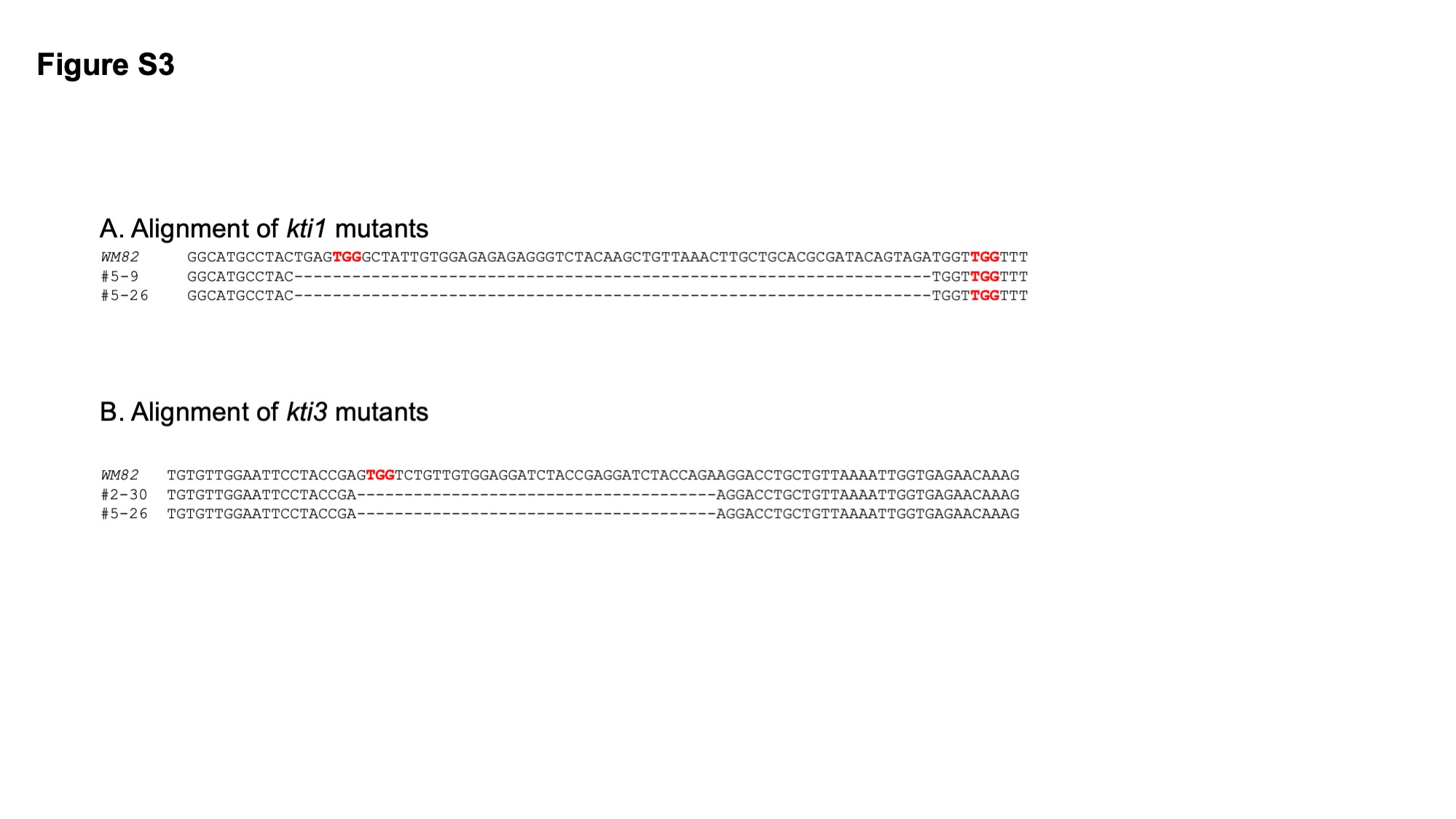

Supplement: Supplementary Figure 3 — Sequence information of KTI1 and/or KTI3 in the T1 plants used for the development of selection markers. (A) The alignment of mutant kti1 in T1 plant leaves (#5-9 and #5-26), where the wild type KTI1 in WM82 was the control. (B) The alignment of mutant kti3 in T1 plant leaves (#2-30 and #5-26), where the wild type KTI1 in WM82 was the control. [file Image_3.jpeg]
